# FAIR assessment of nanosafety data reusability with community standards

**DOI:** 10.1038/s41597-024-03324-x

**Published:** 2024-05-16

**Authors:** Ammar Ammar, Chris Evelo, Egon Willighagen

**Affiliations:** 1 Department of Bioinformatics - BiGCaT, NUTRIM, Maastricht University, Maastricht, The Netherlands; 2 Maastricht Centre for Systems Biology (MaCSBio), Maastricht University, Maastricht, The Netherlands

**Keywords:** Research data, Nanobiotechnology

## Abstract

Nanomaterials hold great promise for improving our society, and it is crucial to understand their effects on biological systems in order to enhance their properties and ensure their safety. However, the lack of consistency in experimental reporting, the absence of universally accepted machine-readable metadata standards, and the challenge of combining such standards hamper the reusability of previously produced data for risk assessment. Fortunately, the research community has responded to these challenges by developing minimum reporting standards that address several of these issues. By converting twelve published minimum reporting standards into a machine-readable representation using FAIR maturity indicators, we have created a machine-friendly approach to annotate and assess datasets’ reusability according to those standards. Furthermore, our NanoSafety Data Reusability Assessment (NSDRA) framework includes a metadata generator web application that can be integrated into experimental data management, and a new web application that can summarize the reusability of nanosafety datasets for one or more subsets of maturity indicators, tailored to specific computational risk assessment use cases. This approach enhances the transparency, communication, and reusability of experimental data and metadata. With this improved FAIR approach, we can facilitate the reuse of nanosafety research for exploration, toxicity prediction, and regulation, thereby advancing the field and benefiting society as a whole.

## Introduction

Nanotechnology is progressively being recognized as a key enabling technology that is helping to remarkably improve many industry sectors and applications like cancer diagnosis^1^, drug delivery^2^, food safety^3^, energy, and environmental science^4^, to name a few. Furthermore, the growing of its importance lead to increase the introduction of engineered nanomaterials (ENMs) to the market^5^. However, as with any new technology, there are also potential risks and uncertainties associated with the use of nanomaterials. These risks and uncertainties arise due to the unique physicochemical properties of nanomaterials, which may result in different biological interactions and toxicological effects compared to their bulk counterparts. Despite the significant efforts to understand the toxicity and safety issues associated with it, nanosafety, is still far from being addressed^6^ and that is partially due to the expensive and time-consuming traditional experimental testing procedures^7^. To address these concerns, the European Commission has funded various research projects aimed at developing a better understanding of the potential impacts of nanomaterials on human health and the environment. From nanomaterial toxicity to exposure monitoring and integrated risk assessment, large amounts of data have been generated for a wide range of nanomaterials. However, not all the data was organized in databases nor sufficient metadata was provided to allow findability and reusability^8^. Moreover, a systemic problem across the field is the inconsistency of standards and experimental reporting. The variability in the published literature regarding the reported experimental and material characterization variables constitutes a significant barrier to progress in such an interdisciplinary field^9^.

In recent years, the concept of FAIR data has been on the rise^10^. FAIR is a set of guiding principles to make the data Findable, Accessible, Interoperable and Reusable. The mindset of the FAIR initiative is of exceptional value for the Nanosafety community and could bring outstanding benefits regarding data standardization, sharing and reuse^11,12^. Data FAIRness refers to the maturation process where digital resources are increasingly becoming self-descriptive to the machine, and hence, facilitating interoperability and reusability of the data among humans and machines at the same time. The FAIR framework is composed of four principles (F, A, I and R) and 14 subprinciples under the main ones (e.g. F1, A2, R1.3), each of which describes an aspect of data FAIRness (see Box 2 in^10^). A limitation of the FAIR framework is the broad textual description of its principles without specifying any technical guidelines on the implementation and the interpretation of those principles, which led to a follow-up work to address this limitation^13^ and another work aimed at defining a way to track and quantify the FAIR aspects of data which was called, maturity indicators^14^. A FAIR maturity indicator (MI) is a measurement that can be used to determine if a digital resource fulfills a particular FAIR (sub)principle^14^, and may give an indication of how the resource can be made more FAIR^15^. Several initiatives proposed maturity indicators definitions ranging from textual descriptions to machine-readable formats. For example, the Research Data Alliance (RDA) developed a data maturity model in textual format^16^. The work developed a standard set of core assessment criteria for FAIRness as an RDA Recommendation. FairPlus is a project with the goal of creating tools and guidelines to enhance the accessibility and reusability of life science data through the FAIR principles. It uses textual descriptions for maturity indicators^17^ and has developed the FAIR cookbook (https://fairplus.github.io/the-fair-cookbook/content/home.html) to guide researchers and data stewards of the life science in their data FAIRification tasks^18^. Wilkinson *et al*.^19^ defined maturity indicators using Markdown and nanopublication formats. Markdown is a markup language used to format plain text (e.g. headings, lists, URLs), and similar to text, it is human-readable. On the other hand, a nanopublication is a machine-readable way of communicating the smallest possible units of publishable information. However, that work excluded from the MI set the subprinciples R1.2 and R1.3 concerning providing rich metadata associated with detailed provenance and meeting domain-relevant community standards, respectively. A common notice of the published literature on FAIR maturity assessment is that evaluating if metadata follows community standards (R1.3) is always absent^11,15,19–21^, as community standards are not formally established yet from the FAIR maturity model perspective.

Providing community standards, of nanosafety, as FAIR maturity indicators under subprinciple R1.3 is a logical progression towards having a harmonized metadata standard and a common ground for nanosafety data FAIRification and reusability assessment. Moreover, capturing structured metadata contributes to the reproducibility and reusability of nanosafety studies, as shown by Elberskirch *et al*.^22^. A Minimum Reporting Standard (MRS), which might also be referred to as a minimum information checklist, a minimum information criteria, a minimum information standard, or a data reporting guideline, defines a set of (meta)data that should be reported by experimentalists and/or captured during data curation^23^. However, the exact metadata that must be reported is highly dependent on the data type and the proposed research question. A review by Stefaniak *et al*. in 2012 identified 28 proposed lists for physicochemical properties that were considered essential for nanomaterial risk assessment^24^. However, physicochemical properties cannot alone determine the risk of nanomaterials and their health impacts. Exposure type, the dose, the tissue/cells in contact, and other variables play a vital role in the toxic effects of nanomaterials due to the intertwined effects of those variables on the biological/environmental behaviour. For example, an inhaled titanium dioxide (TiO_2_) nanomaterial has a different effect than skin contact with cosmetics containing TiO_2_ where studies showed that TiO_2_ did not penetrate the skin to the level of reaching viable cells or the general circulation^25^ while in case of inhalation, it showed a moderate lung inflammation^26^ and nasal irritation^27^ in mice. Therefore, it is important to report variables related to *in vitro*/*in vivo* experimental conditions in nanosafety data to facilitate more effective cross-comparison of experiments and interpretation of their outcomes.

Obtaining reusable data in the nanosafety domain, especially when integrating it from multiple sources, can be better achieved when the metadata complies with a community standard that enables harmonized assessment of data reusability for a particular use case. Moreover, the data should be findable, and its metadata should be expressed in a format that facilitates both the discovery and assessment by humans and machines. In the nanosafety domain, minimum reporting standards were proposed by several teams as a way to facilitate cross-comparison of data and its interpretation^28,29^, support risk assessment^30,31^, assess data completeness^23^, achieve reproducible research^32^, or investigate the environment and health impact of nanomaterials^33,34^. All those MRS developed for different purposes constitute the nanosafety community standards in the broad sense. Making the nanosafety community standards available for FAIR reusability assessment (subprinciple R1.3) in the form of maturity indicators would substantially increase the usefulness of those standards on several levels: (1) provide a reference for researchers and data makers on how to make their data reusable and embed those recommendations within their data workflows and lab notebooks; (2) Re-annotate existing datasets with metadata that complies with the community standards maturity indicators. Thus, already published data would be exposed to all the beneficial applications (see Discussion) of reusability assessment; and, (3) develop software agents that use the nanosafety MIs to assess datasets’ reusability for different use cases.

In this work, we propose a framework for nanosafety data reusability assessment aimed at integrating several minimum reporting standards in the nanosafety domain and using them for data annotation and reusability assessment. We focused in this work on the nanosafety community standards (FAIR subprinciple R1.3). We enabled these standards as maturity indicators that serve two goals: (1) creating machine-readable metadata for nanosafety datasets and (2) assessing nanosafety datasets, accessible over the internet, for reusability for five possible applications. The work also introduced two web applications, one for annotation of data with metadata and the other one to enable automatic assessment of reusability of nanosafety datasets.

## Results

### Identified minimum reporting standards

The search and selection strategy described in the methods section identified 12 sources (Table 1) along with their title, year, DOI and primary use case. Two minimum reporting standards were identified from organizations: the International Organization for Standardization (ISO) and the United Nations (UN). Moreover, two minimum reporting criteria were part of deliverables for EU nanosafety projects, namely, RiskGONE (https://riskgone.eu) and caLIBRAte (http://nanocalibrate.eu). The table shows the number of extracted variables from each source and for which maturity indicators were created. For each extracted variable, a maturity indicator definition was created, including an identifier, provenance information, to which FAIR subprinciple it belongs and several other sections on why and how to measure it, as described in detail in the methods section. In total, 281 maturity indicators were created belonging to the 12 identified MRS lists.

**Table 1:** Selected minimum reporting standards related to nanosafety from literature and the number of maturity indicators created from each one.

Source	Primary Use	# of MIs
Where Are We Heading in Nanotechnology Environmental Health and Safety and Materials Characterization? (2015)^34^ 10.1021/acsnano.5b03496	Nanomaterial properties that play major roles at the nano-bio interface	19
Guidance to improve the scientific value of zeta potential measurements in nanoEHS (2016)^28^ 10.1039/C6EN00136J	Values needed to interpret Zeta-potential meaning and maximize its utility for cross comparison with other reported values	18
Metadata Stewardship in Nanosafety Research: Community-Driven Organization of Metadata Schemas to Support FAIR NanoScience Data (2020)^29^ 10.3390/nano10102033	Nano particle’s agglomeration-related values relevant for delivered dose (DD) assessment as a means to facilitate correct interpretation of *in vitro* bioassays and support data reuse.	37
Minimal analytical characterization of engineered nanomaterials needed for hazard assessment in biological matrices (2010)^33^ 10.3109/17435391003775266	Minimal characteristics and metrics recommended for nanomaterial’s health impact investigations	10
Harmonizing Across Environmental Nanomaterial Testing Media for Increased Comparability of Nanomaterial Datasets (2020)^63^ 10.1039/C9EN00448C	Minimum set of parameters recommended for inter-study comparison of the fate and effects of nanomaterials in biological media	32
Best practice in reporting corona studies: minimum information about Nanomaterial Biocorona Experiments (MINBE) (2019)^32^ 10.1016/j.nantod.2019.06.004	Minimum set of values for the reproducibility of engineered nanomaterials corona characterization studies	28
United Nations Economic Commission for Europe (UNECE) Globally Harmonized System of Classification and Labeling of Chemicals (GHS)^64^	Minimum set of physical and chemical properties to be included in safety data sheets (SDS) of chemicals	19
The Nanomaterial Registry: facilitating the sharing and analysis of data in the diverse nanomaterial community (2013)^65^ 10.2147/IJN.S40722	Descriptors of the Nanomaterial Registry minimal information about nanomaterials (MIAN)’s physicochemical characterization (PCC)	12
caLIBRAte (Nano Risk Governance)^30^ D5.3 Document on quality criteria for data (2017) 10.5281/zenodo.3859951	Recommended parameters to be reported for physical-chemical characterization of NMs and their *in vivo* and *in vitro* human toxicity data	37
RiskGONE toxicity risk data quality measures (2021)^31^	Recommended set of *in vivo* and *in vitro* parameter for toxicity risk assessment data	34
ISO/TR13014 Nanotechnologies – Guidance on physio-chemical characterization of engineered Nanoscale materials for toxicological assessment (2012)^66^	Physico-chemical characteristics that have been proposed as the most relevant to toxicological assessment	9
Minimum information reporting in bio–nano experimental literature (2018)^9^ 10.1038/s41565-018-0246-4	minimum information standard for experimental literature investigating bio–nano interactions	26

#### The scope of the NSDRA maturity indicators

The developed NSDRA maturity indicators provide guidance about what should be included in a nanosafety-related dataset, set a specific format, and define a schema of how to represent the metadata of these datasets in an interoperable machine-readable way. Moreover, we believe that adopting the linked-data formats for data sharing and reuse will further advance the field of nanosafety by promoting the curation and collection of high-quality data, facilitating data completeness assessment and enabling data-driven modeling. While there are many different assays and techniques available for measuring the variables defined in our maturity indicators, it is not our intention to enforce specific protocols or assays, or highlight their relative advantages or disadvantages. However, reporting the experimental assays and protocols used is critical for ensuring the reusability of nanosafety data. We recognize that defining nanosafety community-related metadata standards in compliance with the FAIR subprinciple R1.3 should not add significantly to the experimental workload routinely required in this field. Indeed, many of the variables and parameters we defined are measured or calculated by default, and reporting them in the data and metadata should not pose a significant burden. Finally, our approach also places special emphasis on reporting standards that include both characterization properties for nanomaterials and *in vitro*/*in vivo* experimental variables. This emphasis underscores the importance of these variables and encourages researchers to include them in their datasets. By following these reporting standards, researchers can ensure that their data is more transparent, reusable, and ultimately contributes to advancing the field of nanosafety.

### Content analysis of nanosafety community-standards maturity indicators

**Fig. 1:**
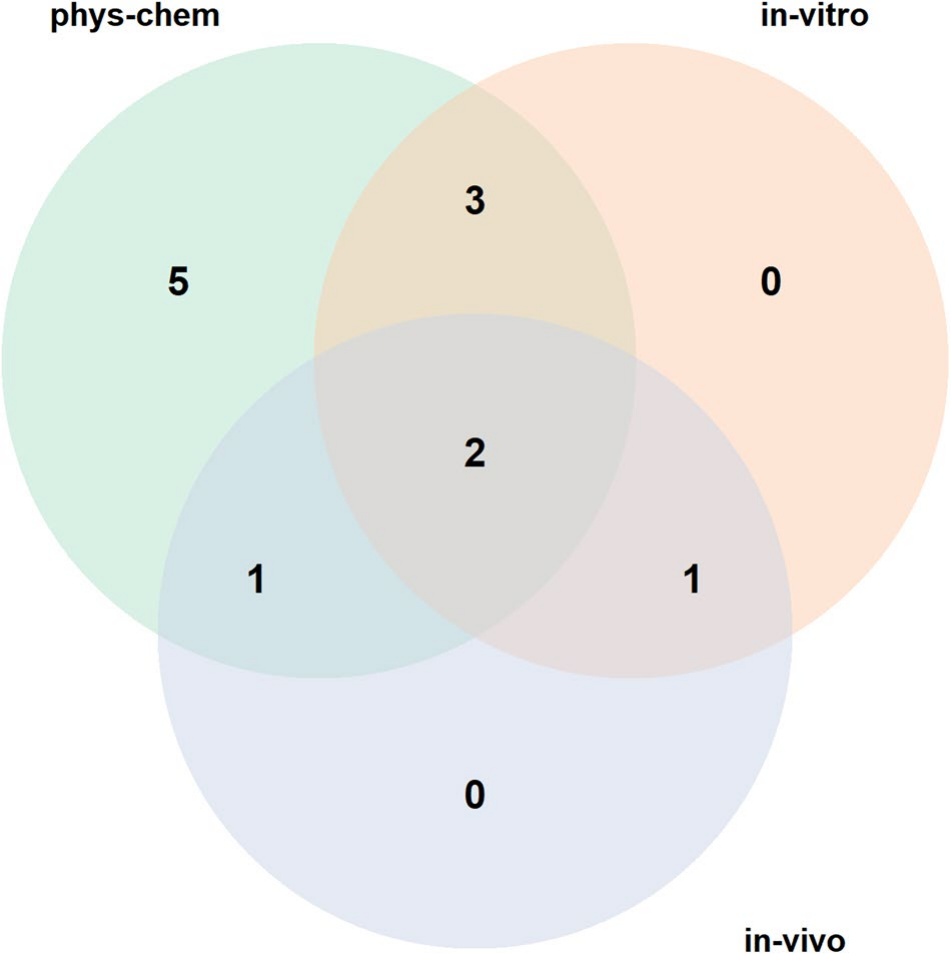
Venn diagram showing the coverage of the minimum reporting standards (MRSs) over three groups of measured variables (physicochemical characterization, *in vitro* and *in vivo*). The figure shows that five MRSs are dedicated to reporting variables related to the physicochemical properties of nanomaterials, while two MRSs included variables related to all three groups of variables. Moreover, none of the MRSs were dedicated to *in-vivo* or *in-vitro* variables alone.

The 12 lists of maturity indicators listed in Table 1 show variability in the number of variables to be reported depending on their coverage and level of detail, where 9 is the lowest number of maturity indicators a list has, and 37 is the highest. The MIs, defined as Java properties files, are available in a GitHub repository. Also, another GitHub repository has the generated markdown and nanopublications for MIs. In an attempt to analyze the content of the MI lists as described in the methods section, the Venn diagram in Fig. 1 shows the content coverage of the MI lists by grouping their MIs into three groups representing their nature (physiochemical, *in vitro*, and *in vivo*). It shows that five lists contain only physicochemical characterization-related maturity indicators. In contrast, none of the lists is dedicated to *in vivo* or *in vitro* MIs, and only one list contains both *in vitro* and *in vivo* MIs without physiochemical-related ones. Moreover, three lists cover physicochemical and *in vitro* MIs while only one list covers physicochemical and *in vivo* MIs. Finally, two lists cover all of the three categories of maturity indicators. The undergone effort of grouping the maturity indicators into generic indicators and mapping those to five nano-related applications in Table 2 allowed better comprehension of the coverage of the 12 MI lists and their possible use-cases. The 30 generic indicators grouped 119 overlapping maturity indicators from the 12 lists. Also, lists 9 and 10 (caLIBRAte and RiskGONE), have the most coverage across the three groups of indicators (basic, physicochemical and toxicity). Another observation from the table is that no single MI list fulfills all of the applications and often annotations from more than one list are needed to assess for full compliance with a target application. Furthermore, list 5 does not have any indicator that can be grouped under the generic indicators due to the specific purpose of this list towards harmonising nanomaterials testing media, and thus, it does not provide indicators related to toxicity or physicochemical properties. Supplementary Table S1^35^ shows a breakdown of the MIs grouping into the generic indicators along with links to the Markdown description of each maturity indicator on GitHub.

**Table 2:** The table shows three groups of variables with maturity indicators belonging to at least two MI lists linked to them.

Group	Variable	Maturity indicators lists												Applications				
		01	02	03	04	05	06	07	08	09	10	11	12	GR	NI	TP	RR	NC
Basic	Chemical composition	x	x		x				x	x		x	x	x	x	x	x	x
	Surface chemistry/coating	x	x				x		x	x		x		x	x	x	x	
	Purity	x			x				x	x	x			x		x		
	Nanomaterial labeling/identity										x		x					
	Nanomaterial source									x	x							
PhysChem	Size	x	x	x	x				x	x		x	x	x	x	x	x	x
	Shape	x	x	x					x	x		x	x	x	x	x		x
	Surface Area	x			x				x	x		x		x	x			
	Surface charge	x		x	x				x	x		x		x		x		
	Zeta potential	x											x	x		x	x	
	Crystallinity	x								x				x	x			x
	Solubility				x			x	x			x		x		x		
	Stability								x	x								
	Dispersibility			x								x		x				
	Density			x				x					x					
	Agglomeration state	x					x		x	x		x						
Toxicity	Dose/Concentration		x	x						x	x		x			x		
	Exposure duration/time			x						x	x			x		x		
	Number of controls									x	x			x		x		
	Number of replicates		x				x				x			x		x		
	Data analysis methods										x		x	x		x		
	Organism/Species									x	x			x		x		
	Method/route of administration									x	x		x					
	*in vivo* - Number of test subject									x	x							
	*in vivo* - Subject age									x	x							
	*in vivo* - Subject sex									x	x							
	*in vivo* - Subject weight									x	x							
	*in vivo* - Subject strain									x	x			x		x		
	*in vitro* - Passage number									x			x					
	*in vitro* - Cell mycoplasma testing									x			x					

The variables are mapped to five applications (GP: Grouping/Read-across, NI: Nanoform Identification, TP: Toxicity Prediction, RR: Regulatory requirements, NC: NanoInChI Calculation) determined by experts in the nanosafety domain showing variables deemed important to be reported for each application. The table visualizes the overlap between the MI lists and their link to potential applications.

### NSDRA JSON-LD metadata generator

**Fig. 2:**
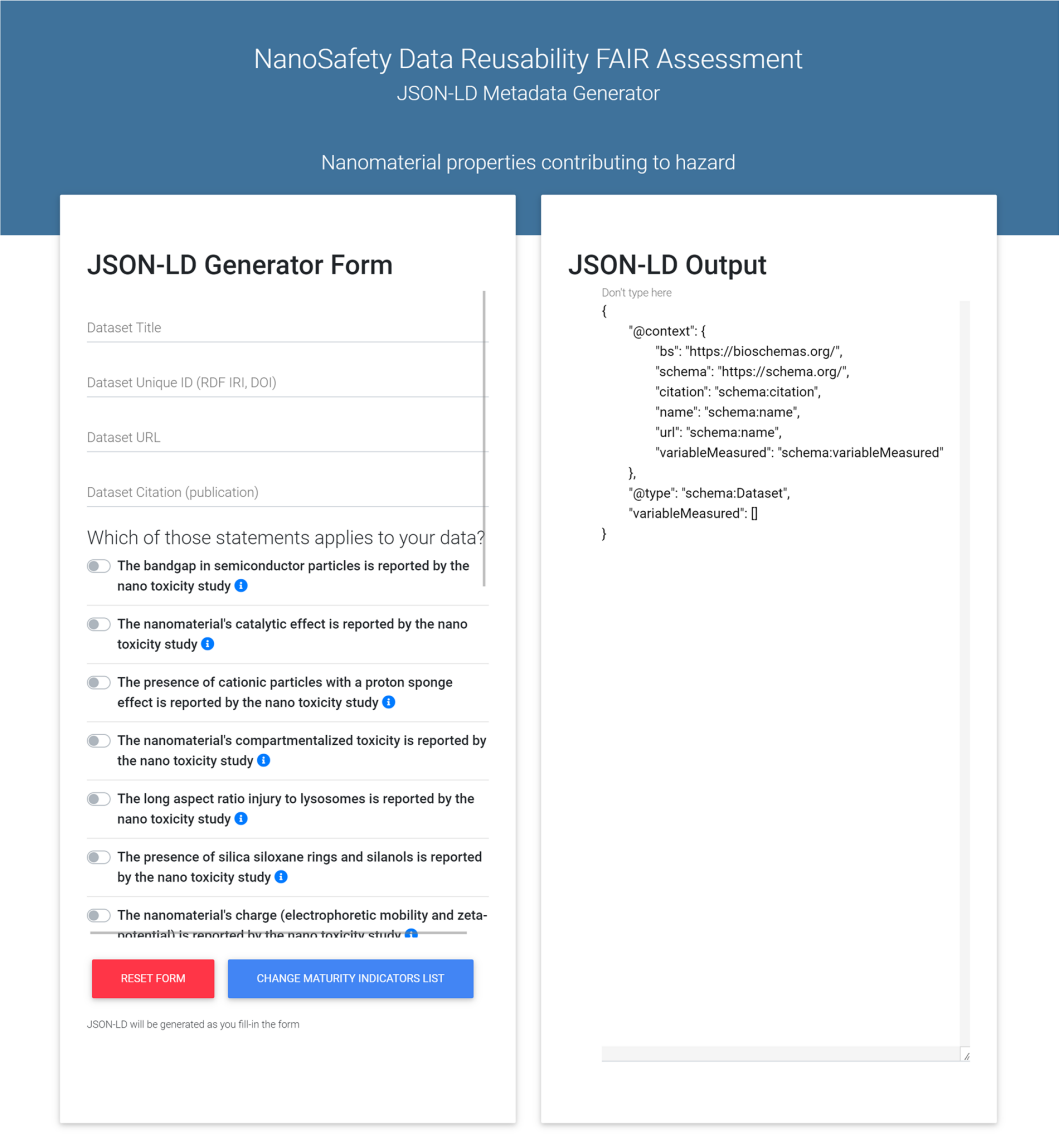
A screenshot of the NSDRA JSON-LD metadata generator. In the left panel, the variables of the minimum reporting standard, which are defined as FAIR maturity indicators, are listed with a check box next to each one. When the user checks each variable to be included in the metadata (i.e. the variable is reported in the dataset), the corresponding JSON-lD metadata annotation is automatically generated in the right panel of the web application interface.

The metadata generator^36^ was developed to help users create JSON-LD metadata for their data. The web interface of the application starts with showing the available list of minimum reporting standards. The user chooses the preferred one for describing the data. Next, a web form is shown (Fig. 2) where the user fills the fields, and JSON-LD metadata is automatically generated. The interface (Fig. 2) has two panels. The left panel contains the form, which requires two types of input. At the top, there are four text fields (Dataset title, unique ID, URL, and citation) to record the provenance information of the dataset. Beneath that, there is a list of check boxes corresponding to the variables to be reported by the chosen minimum reporting standard. Practically, a maturity indicator describes each reported variable. Hence, ticking a box implies that the variable described with this maturity indicator is reported in the described dataset. Next to each tick box, there is an information icon link that takes the user to the markdown description page of the maturity indicator to learn more and make sure the choice is valid. On the other hand, the right panel contains the generated JSON-LD, which can be copied by the user and used anywhere on the web, like embedding it in the web page that hosts the dataset (data repository, personal website, institution website). The web application is available for use (http://w3id.org/nsdra/metadata-generator), and the code is available on GitHub.

### NSDRA web application for automated completeness and reusability assessment

**Fig. 3:**
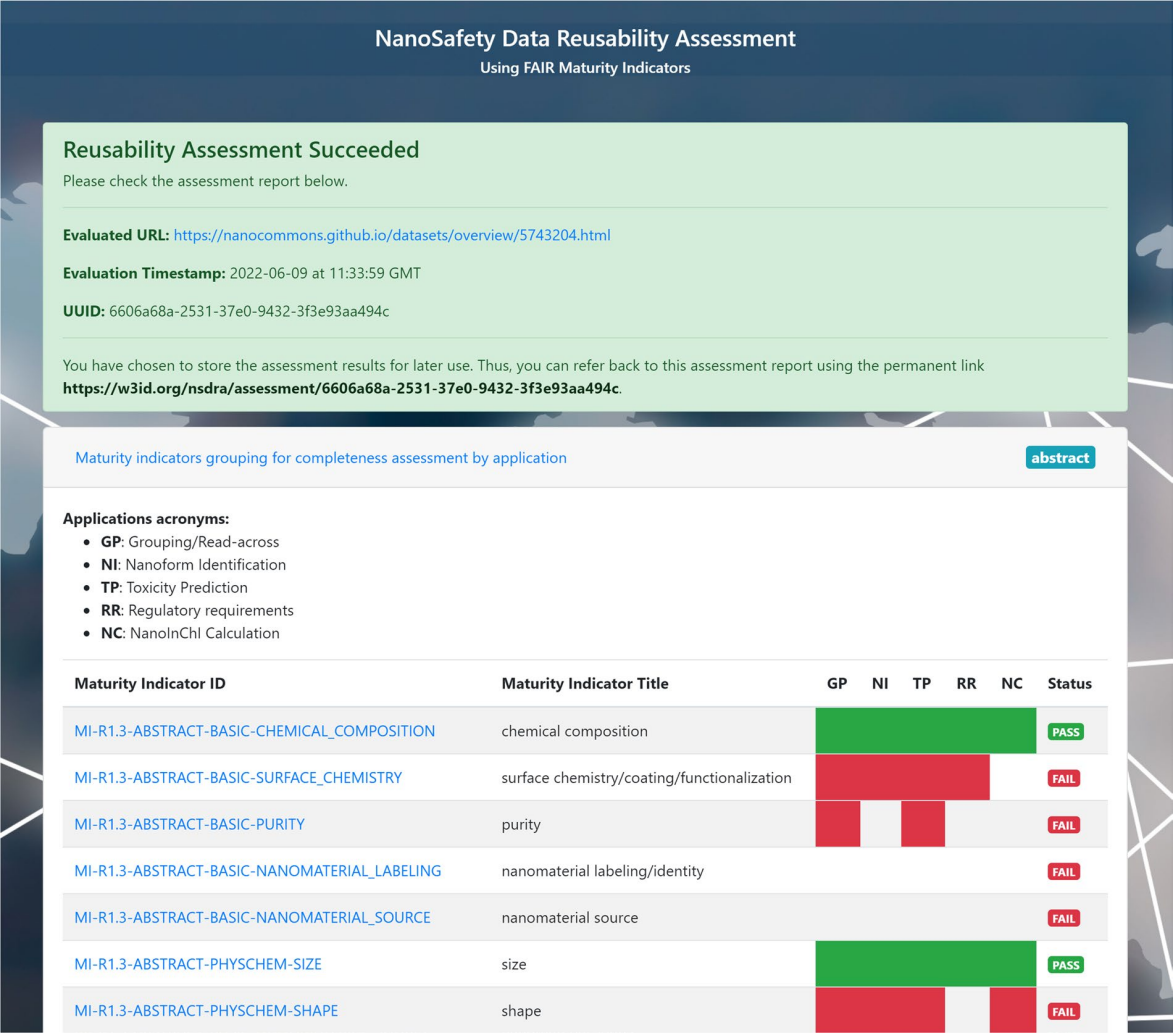
A Screenshot of the NSDRA reusability assessment web application showing the interface of the application after evaluating a nanosafety dataset against an MI list. The screenshot shows in the top panel a summary of the URL submission for assessment including timestamp, assessment status, and the globally unique identifier for the assessment report. The bottom panel shows the assessment results. For this particular example, the target URL is assessed against the generic set of maturity indicators where a pass badge is present if the corresponding variable appears in the machine-readable metadata that is extracted from the URL, and a fail badge is present if the variable is absent from the metadata.

NSDRA web application^37^ is accessible through https://nsdra.org. Users provide a URL for the resource to be assessed which contains JSON-LD metadata in its markup. Next, the user chooses the maturity indicators from a multiple choice checkbox list against which the digital resource will be assessed. Users can also choose to save the results of the assessment and get a unique URL for the assessment report recording the timestamp of the assessment, the assessed URL and the results. Such a resource can be used for reference purposes or for self-assessment and continuous improvement of nanosafety data reusability. The assessment using the web application was applied on five dataset overview pages from the catalog of open datasets released by the NanoSafety Cluster (https://nanosafetycluster.eu) project against the caLIBRAte MI list. Figure 3 shows a screenshot from the assessment page of the test URL https://nanocommons.github.io/datasets/overview/5743204.html. We chose to save the assessment report where a persistent URL is minted as a reference. The saved assessment can be viewed on this URL: https://w3id.org/nsdra/assessment/ea1c58fd-0b13-3a04-9a04-87c87b88659e.

## Discussion

We proposed a framework to standardize, facilitate and assess data reusability in the nanosafety domain from the FAIR perspective. The framework comprises 12 lists of maturity indicators derived from corresponding minimum reporting standards, a web application to help users generate JSON-LD metadata for their data that comply with the nanosafety maturity indicators (i.e., community standards), and a web application for data reusability assessment. The assessment application can assess any web page containing machine-readable metadata that complies with the maturity indicators defined in the work.

The selection of the articles focused on the ones after 2010 to keep up with the latest efforts in defining reporting standards. The selected sources recommended values to report were divided into three groups (physiochemical, *in vitro*, and *in vivo*), and their coverage was analyzed using a Venn diagram. Building the maturity indicators on top of already published standards and guidelines was deliberate for two reasons. First, to increase the adoption of the proposed framework since the underlying components were already reviewed and accepted by the community. Second, to enforce those standards by providing a framework that allows users to generate machine-readable metadata that complies with community standards.

The JSON-LD format was used to enforce machine-readable metadata, allowing software agents to parse and understand the metadata. Moreover, using the schema.org vocabulary allows the annotated resource to be discoverable by search engines like the Google dataset search^38^ that will recognize and index any web page that uses the schema.org “Dataset” annotation. However, learning JSON-LD needs time and dedication, which may not be feasible for all researchers, especially those from a different domain like wet-lab experimentalists. Therefore, a web application was developed to allow researchers and data creators in the nanosafety domain to create metadata for their datasets in JSON-LD, which can be embedded in any web page. With such flexibility being provided, discoverable datasets are not limited to data repositories and registries. However, they can be extended to any form of websites such as personal blogs, tutorials, courses, and institution websites. It is worth mentioning that some online repositories like Figshare already use JSON-LD as a metadata representation format for the datasets published on that platform. The metadata also uses the schema.org vocabulary and the Dataset class annotation.

We did our best to ensure the FAIRness of all of the components of the framework. First, all the code is made available under open-source license on GitHub. The repositories were preserved using Zenodo, and a DOI was minted for each one of them, which keeps track of versioning. The MIs have nanopublications associated with them with persistent identifiers through the w3id.org organization. The assessment reports themselves are also assigned globally unique identifiers using w3id.org URLs and UUID generated by the NSDRA assessment web application. Moreover, the assessment web page is annotated with schema.org “Review” entity, and software agents can parse the report and extract lists of passed and failed tests along with provenance information about the assessed resource. Adopting the framework described here can be advantageous in several ways, including (1) Aiding in data curation efforts like the Nanomaterial Data Curation Initiative (NDCI)^39^ and constructing big databases like PubVinas^40^. In such scenarios, identifying the coverage and the content of a dataset in an automated way can increase efficiency and quickly identify good candidate data sources and categorize them into groups for the next step in the curation process. (2) Data-driven modeling of nanotoxicology^41^ and Risk assessment strategies^42^. In those cases, it is of utmost importance to know what variables have been measured in the available data source and guide the selection process to build up the dataset for the modeling approach. For example, one can check for the datasets that have measured the targeted output variable and select only those for supervised learning model development. (3) Facilitate integration among databases and datasets^43^, which can be pushed forward by adopting a unified vocabulary to represent metadata. It will help overcome the challenges of using different terminologies and formats to represent metadata. Moreover, it allows integration with data sources from other disciplines using the same metadata format and vocabulary. Also, it helps to combine knowledge to gain better insights and facilitate deeper data analysis. The NSDRA framework is intentionally not coupled with any data repository or provider. The aim is to be able to generate machine-readable metadata, adopting open standards, for any dataset without being bound to a specific database or vendor requirements. For example, Basei *et al*.^44^ proposed an integrated tool into eNanoMapper^45^ for the automatic evaluation of data quality and completeness of nanomaterials for risk assessment purposes. (4) Enhancing and developing new material safety data sheets. Eastlake *et al*.^46^ have shown that 67% of the material safety data sheets (MSDS) obtained in 2010–2011 still provided insufficient data for communicating the potential hazards of engineered nanomaterials. Using the community standards in the nanosafety domain and an interoperable representation format can substantially benefit developing MSDSs. For example, tools can be developed to retrieve machine-readable MSDS released by governments and regulatory bodies to be used by industries in selecting ingredients for their products or by inspectors to check the compliance of industrial products with minimum safety standards. (6) Support quality evaluation and data completeness frameworks and platforms^23,47^ (e.g.: GUIDEnano^48^). Checking for data quality and completeness is an inevitable step for any risk assessment or nanotoxicity modeling task. Checking for datasets that comply with community standards regarding their measured variables and the ability to do that in an automated way is a clear advantage and a valuable feature to have in such platforms and frameworks.

For future work, several improvements can be made to the proposed framework. For example, the current framework supports only JSON-LD format, which can be extended to other semantic formats like RDF. Moreover, the maturity indicators definition can be extended to cover the units and missing values of the measured variables. For example, a field can be added to the JSON-LD schema for each measured variable with a boolean value. If there are missing values in the measure variable’s column in the dataset, then the value is TRUE. Otherwise, it is false. Introducing these additions will take the framework to a new level where it can be actively used for automated data completeness assessments. Finally, currently, the maturity indicators are simple yes/no tests that check if the variable is reported or not. However, this can be extended to more complex ones (like specific experimental conditions combining multiple variables or platforms).

## Methods

### Data sources

#### Selection of articles

Identifying the minimum reporting standards and best practices related to the nanosafety domain in literature is a multi-step process that was manually performed to obtain the most suitable sources. First, a literature search for potentially relevant articles published between 2010 and 2021 was conducted through PubMed (https://www.ncbi.nlm.nih.gov/pubmed), Google Search Engine (https://google.com), and Europe PMC (https://europepmc.org)^49^. Keywords, such as “minimum reporting standards”, “nanomaterial”, “characterization”, “best practices”, “guidelines”, “metadata”, “data quality”, “data completeness”, “reusability”, “nanosafety” were used in combination to form multi word search queries. Second, judging by the title and abstract, relevant articles mentioning, defining, assessing, listing nano-related properties, parameters, characteristics or standards required to assess the safety or maximize the utility of data were retrieved for further assessment. Third, bibliographies of the relevant articles and the Google Scholar profiles of their authors were screened for more references. Also, standards and technical reports from organizations were retrieved and examined whenever mentioned in the text. In the end, the final selection of sources was determined by assessing the following inclusion criteria: (1) published peer-reviewed articles introducing widely applicable reporting standards and not targeting a specific dataset or nanomaterial; (2) the minimum values/parameters to be reported are presented in a structured way (figures, tables, supplementary materials) and not just scattered in the text; (3) the MRS covers one or more of the following three groups (physicochemical characteristics, *in vivo* and *in vitro* experiment parameters); (4) the sources published in English; and (5) published after 2010 (including). The decision to search for articles published after 2010 was mainly to cover the most recent standards in the field and stay up-to-date with guidelines and best practices. However, several checklists and MRSs were published before 2010 and already referenced in the selected sources.

### Maturity indicators definition workflow

**Fig. 4:**
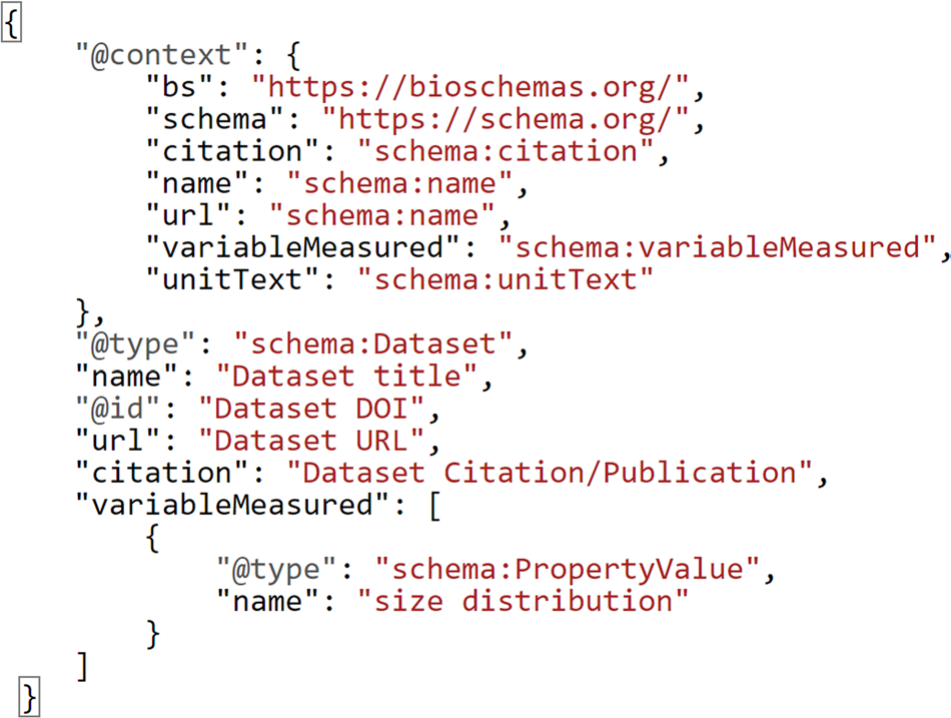
JSON-LD snippet of a good example to report measured variables metadata for a nanosafety dataset using the schema.org vocabulary.

A maturity indicator is a measurable aspect of a FAIR (sub)principle that evaluation approaches can use. From this definition, it can be inferred that a maturity indicator should describe a self-contained aspect of the (meta)data and provide information on how it should be represented, measured, and assessed from the FAIR point of view. The definition of a maturity indicator can be implemented in different ways, from textual descriptions to semantic formats that both humans and machines can consume. Since this work aimed at automating the process of reusability assessment using maturity indicators, it was of great importance to choose a machine-readable definition format. Moreover, we considered two designs when defining the maturity indicators. First, the metrics themselves and the applications developed around them should be FAIR. Second, they should stay up to date with proper versioning and provenance information. For the reasons mentioned earlier, Wilkinson *et al*.^19^ implementation was chosen as the base to express the selected lists of MRS as reusability maturity indicators (under FAIR principle R1.3). However, whenever needed, it was adapted to accommodate the requirements of the nanosafety domain. In the original specification, the user fills the maturity indicator’s Markdown template, which can be automatically converted to the nanopublication version using a specific software. However, this approach was adjusted in this work, so the Markdown template is populated with the necessary fields from a simple key-value text file, called a properties file. This file serves as a template to fill in the necessary information when defining each maturity indicator. Later, this properties file can be converted to any other template like Markdown or nanopublication. Another convention was enforced in the MI definition process regarding what must be provided for the measurement and how the measurement is executed. Originally, those criteria were flexible and can be described in any way by the user, who can later provide a coded compliance test to perform the evaluation. However, in this work, JSON-LD was explicitly chosen as the representation format of the assessed digital resource’s metadata. That means, if the digital resource does not use JSON-LD format to represent the metadata, then it will not be qualified to pass the reusability evaluation for any of the maturity indicators. JSON-LD is a lightweight linked data format to easily read and write structured data on the web using open vocabularies like schema.org^50^ and bioschemas.org^51^. In other words, JSON-LD is understandable by the machine, and adopting it by data providers to express metadata allows an automated agent to locate, parse and assess the content of the metadata without human intervention. Figure 4 shows a JSON-LD snippet of valid metadata expressing the measured variable/parameter using the “variablesMeasured” property of the schema.org “Dataset” Class.

**Fig. 5:**
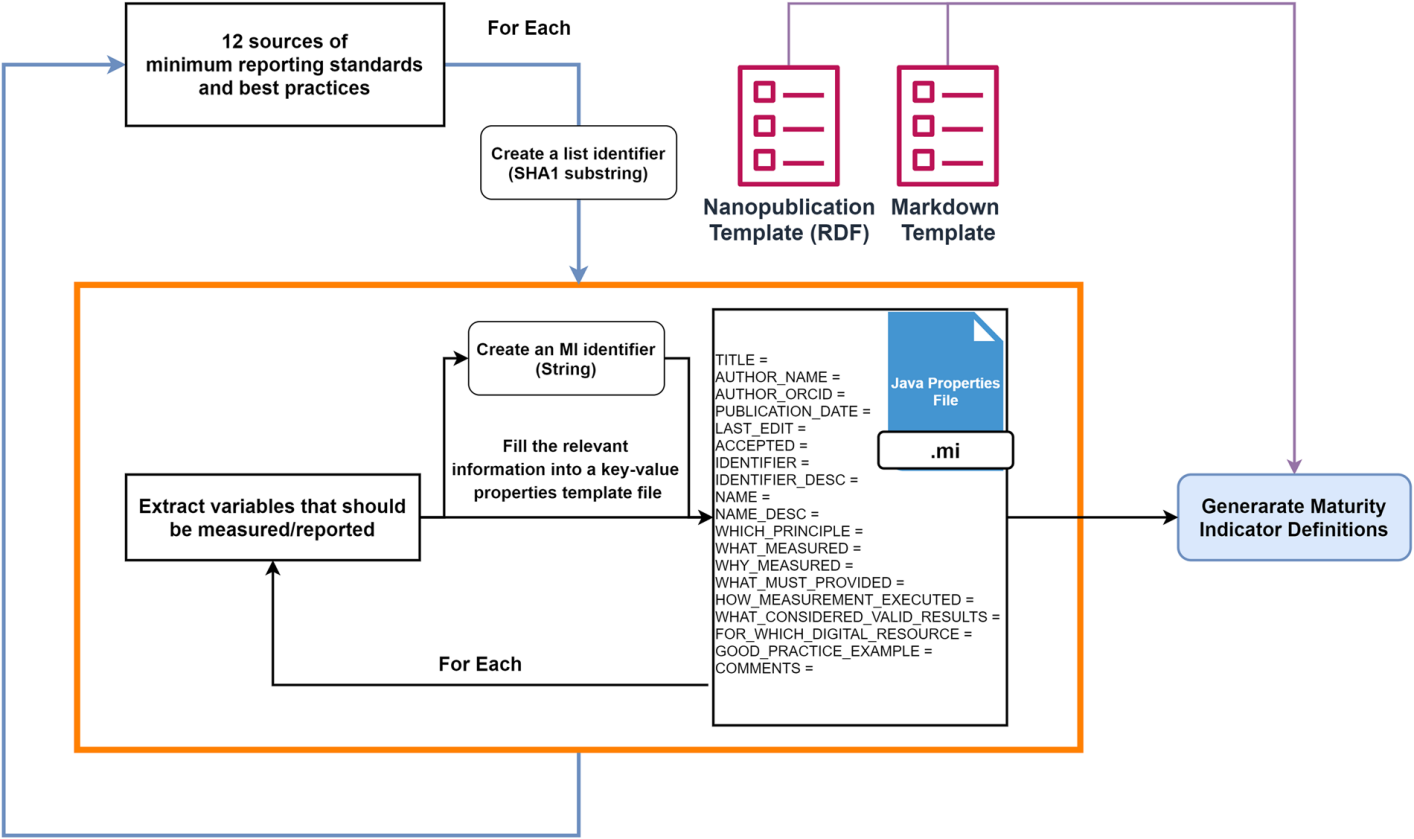
Maturity indicators definition workflow. The workflow starts with a loop over the selected source. Next, for each source, the variables are extracted from the source and described using a key-value file format derived from Wilkinson *et al*., which are then converted into markdown and nanopublication formats using an in-house developed Java tool.

The workflow of defining maturity indicators, as depicted in Fig. 5, starts with the identified sources of minimum reporting standards. Next, for each source, a unique identifier is created to identify the list of maturity indicators that will be created from it. The identifier is created by computing the SHA256 hash of the DOI if the source is a publication or the URL of the main website of the source otherwise. Next, the first ten characters of the hash are used as a list identifier. Next, the source is thoroughly examined to identify the variables/parameters that should be measured or reported and thus should be described as maturity indicators. After that, an identifier for each candidate maturity indicator is created according to the following template: MI-R1.3-LIST_IDENTIFIER-INDICATIVE_STRING. For example, in the following identifier “MI-R1.3-649848907b-MEDIA_PH”, the list identifier is “649848907b,” and the indicative string is “MEDIA_PH.” Next, the information related to the variable is extracted from the source and provided as a simple key-value text file, called a properties file. The process is repeated for each variable and each source. Finally, an in-house developed Java tool is used to convert the properties files into the final formats of Markdown and nanopublication. The generator of the MIs from the properties file is a command-line tool written in Java and also hosted on GitHub. The tool can be used by executing the following command from terminal:

$ java -jar MI-gen-v1.0.jar -s SOURCE_PATH -d DEST_PATH

SOURCE_PATH: is the path of the maturity indicators defined as Java properties files. DEST_PATH: is the path where the generated maturity indicators (Markdown & nanopublication) will be placed, along with a JSON file describing the lists (needed for the JSON-LD generator web app). Moreover, the tool performs checks to: (1) validate the Trig syntax of the nanopublication using RDF4J^52^. (2) validate the nanopublication format using the nanopub-java library^53^. (3) generates Trusty URIs^54^ for the nanopublication format using the trusty-java library which is required to publish them on the decentralized network of nanopublications. Following the previous process, the MI lists were tagged with three groups capturing the nature of their content: physiochemical, *in vivo*, and *in vitro* maturity indicators, and a Venn diagram was produced to depict their coverage.

### Maturity indicators grouping

In order to maximize the utility of the defined maturity indicators, they were mapped to a generic set of variables in such a way that overlap between the 12 lists can be observed and quantified. For example, maturity indicators related to nanomaterial size were grouped together and that includes: diameter, aspect ratio, dimensions, and size distribution. Moreover, the generic set of indicators was also mapped to five different applications in the nanosafety domain highlighting the minimal reporting of variables needed for each of those applications, as shown in Table 2. The mapping between the generic variables and the five applications was reused from a table provided in the NanoSolveIT EU project deliverable report D1.7^55^. Originally, the initial mapping between variables/endpoints and the five applications was based on reviews published by GRACIOUS^47^ and the ToxRTool quality measures^56^ as well as the NanoInChI concept^57^ and the ECHA guideline on QSAR information requirements and read-across^58^.

### NSDRA metadata generator

As described in the previous section, JSON-LD was determined to be the metadata format required for the automated evaluation of the digital resource. However, data providers and publishers like wet-lab experimentalists and researchers in the nanosafety domain may not be familiar with this format’s technical details. Moreover, learning and practicing its use for daily metadata annotation could be difficult and time-consuming. For the reasons mentioned above, a web application was developed to generate metadata complying with the MRS of choice in JSON-LD format without the need to understand the details of that format. This application is complementary to the maturity indicators within the proposed framework. Then, the generated metadata can be submitted to data repositories, databases, data registries, or any URL on the internet with or without the data. This way, any resource URL that has the generated metadata is qualified for the usability assessment carried out by automated applications. NSDRA metadata generator^36^ is a javascript-based front-end module that dynamically generates form-based web interfaces from a maturity indicators list JSON object. It is able to generate the web form on the fly corresponding to the user’s choice of one of the defined maturity indicators lists (i.e., community standards). Moreover, while the user fills out the form fields, a valid JSON-LD markup is automatically generated and updated with each user interaction with the form. The generated JSON-LD is machine-readable FAIR-compliant metadata that can be automatically assessed using the NSDRA web server application or any other evaluator software capable of consuming JSON-LD format. The app is built using modern web technologies and frameworks HTML5^59^, CSS3^60^, Bootstrap 4, and JQuery. The main aim of this app is to assist users, especially experimentalists and wet-lab researchers who have minimal knowledge of semantic web formats, in creating their metadata structure and allowing easier development of linked data-compliant markup for data resources.

### NSDRA web application for automatic assessment

The NSDRA application^37^ is a web application written in Java under Spring Framework, with source code available through GitHub. It utilizes BMUSE^61^, a scraping framework capable of extracting JSON-LD and RDFa markup from static and single-page application sites. BMUSE was originally developed to scrape and extract Bioschemas markup. The NSDRA application is primarily designed to read the 12 maturity indicator lists from a GitHub repository and apply them to the user-provided nanosafety dataset (as a URL describing the dataset) for evaluation. The application scrapes the provided URL, extracts JSON-LD embedded in its HTML, and converts it to in-memory RDF to apply the reusability MI tests on it. Moreover, the application allows the user to register the evaluation results in a database for later retrieval or reference purposes. Figure 3 in the Results section was generated using the mentioned web application, which is currently deployed on a server (32GB RAM and 4 CPUs) and running on a Tomcat web server, with evaluation results persistence handled by MySQL 8.0 database server. The web application requires Java 8 at least to operate and can be deployed using a Docker container. To test the web application, five dataset overview pages (annotated with JSON-LD) from the catalog of open datasets released by NanoSafety Cluster projects https://nanocommons.github.io/datasets/ were used for assessment. The selected pages provide an overview of five nanosafety-related datasets published on Zenodo and also annotated, as part of this work, with variables measured according to the maturity indicators of MI list 10 (caLIBRAte criteria). Thus, their annotation is machine-readable and can be assessed using the NSDRA web application. The assessor application fetches the URL of the dataset overview page, scrapes its content and identifies the JSON-LD markup. Next, using the JSON description of the maturity indicators, the assessor locates the reported measured variables, compare them to the MI list of choice and reports the matching variables.

### The FAIRification of maturity indicators

The Java converter tool mentioned earlier parses the MI properties file and converts it to Markdown and nanopublication formats similar to the work of Wilkinson *et al*. and thus making it interoperable with the output of that work. Also, the nanopublications are assigned a Trusty URI which is required to publish them to the nanopublications network. The Trusty URI uses a base URL from w3d.org which makes it a persistent identifier. Moreover, the NSDRA metadata generator, MI generator, and the evaluator web application are provided through open source GitHub repositories and preserved through the Zenodo platform with a DOI minted for each one. This way, the findability and accessibility are achieved, and the versioning, since Zenodo keeps track of GitHub versions and assigns a DOI for each new version. Using an open license and the semantic representation of the maturity indicators supports interoperability and reusability and adds to the FAIRness of the framework^62^.

### Supplementary information

Supplementary materials

## Data Availability

All the maturity indicator definitions in both Markdown and nanopublication formats are available online in GitHub (https://github.com/NSDRA) and archived on Zenodo with: https://zenodo.org/doi/10.5281/zenodo.10886195.

## Code availability

The code for the JSON-LD metadata generator and the NSDRA reusability assessment are available online in GitHub (https://github.com/NSDRA) and archived on Zenodo with the following DOIs: NSDRA Metadata Generator Web Application: 10.5281/zenodo.10886178 NSDRA Reusability Assessment Web Application: 10.5281/zenodo.10886180.
