# Supplementary material for: FAIR assessment of nanosafety data reusability with community standards
Source: Sci Data. 2024 May 16;11:503. doi: 10.1038/s41597-024-03324-x (PMC11099147; doi:10.1038/s41597-024-03324-x)
Supplement: Supplementary file 1 — Supplementary materials [file 41597_2024_3324_MOESM1_ESM.pdf]

FAIR assessment of nanosafety data reusability with community standards

Ammar Ammar[*1], Chris Evelo[1,2] and Egon Willighagen[1*]

[1*]Department of Bioinformatics - BiGCaT, NUTRIM, Maastricht University, The Netherlands.
[2]Maastricht Centre for Systems Biology (MaCSBio), Maastricht University, The Netherlands.

*Corresponding author(s). E-mail(s):
a.ammar@maastrichtuniversity.nl;
egon.willighagen@maastrichtuniversity.nl;
Contributing authors:
chris.evelo@maastrichtuniversity.nl;

Supplementary Materials

Table S1: Grouping similar maturity indicators by general variables. The similar maturity indicators (i.e. overlapping in what they measure) from more than one maturity indicator list are grouped by generic variables.

Variable	MI List	Maturity Indicator
passage number	09-7a4d616c66	MI-R1.3-7a4d616c66-IN_VITRO_SUBJECT_PASSAGE_NUMBER
	12-d2c4887a02	MI-R1.3-d2c4887a02-BIO_PASSAGE_NUMBER
number of test subject	09-7a4d616c66	MI-R1.3-7a4d616c66-IN_VV_METHODS_NUM_OF_SUBJECTS
	10-57299b68d4	MI-R1.3-57299b68d4-IN_VIVO_NUMBER_OF_SUBJECTS
Subject weight	09-7a4d616c66	MI-R1.3-7a4d616c66-IN_VIVO_SUBJECT_BODY_WEIGHT
	10-57299b68d4	MI-R1.3-57299b68d4-IN_VIVO_ORGANISM_WEIGHT
Subject age	09-7a4d616c66	MI-R1.3-7a4d616c66-IN_VIVO_SUBJECT_AGE
	10-57299b68d4	MI-R1.3-57299b68d4-IN_VIVO_ORGANISM_AGE

**Subject sex**	09-7a4d616c66	MI-R1.3-7a4d616c66-IN_VIVO_SUBJECT_SEX
	10-57299b68d4	MI-R1.3-57299b68d4-IN_VIVO_ORGANISM_SEX
**cell mycoplasma**	09-7a4d616c66	MI-R1.3-7a4d616c66-IN_VITRO_SUBJECT_MYCOPLASMA_TESTING
	12-d2c4887a02	MI-R1.3-d2c4887a02-BIO_MYCOPLASMA_TESTING
**agglomeration state**	01-75ec3968cc	MI-R1.3-75ec3968cc-EXTRINSIC_STATE_OF_AGGLOMERATION
	06-324358ad68	MI-R1.3-324358ad68-I-AGGREGATION_STATE
	08-3ce932c4f9	MI-R1.3-3ce932c4f9-AGGLOMERATION_STATE
	09-7a4d616c66	MI-R1.3-7a4d616c66-PCHEM_AGGLOMERATION_STATE
	11-1fb3ef13d0	MI-R1.3-1fb3ef13d0-PCHEM-AGGLOMERATION_STATE
**density**	03-88b52fa7b8	MI-R1.3-88b52fa7b8-PARTICLE_DENSITY
	07-1582738113	MI-R1.3-1582738113-PCHEM-DENSITY
	12-d2c4887a02	MI-R1.3-d2c4887a02-MAT-DENSITY
**nanomaterial labeling/identity**	10-57299b68d4	MI-R1.3-57299b68d4-IN_VITRO_SUBSTANCE_IDINTITY
	10-57299b68d4	MI-R1.3-57299b68d4-IN_VIVO_SUBSTANCE_IDINTITY
	12-d2c4887a02	MI-R1.3-d2c4887a02-MAT-LABELING
**Nanomaterial source**	09-7a4d616c66	MI-R1.3-7a4d616c66-IN_VV_SUBSTANCE_NP_SOURCE
	10-57299b68d4	MI-R1.3-57299b68d4-IN_VITRO_SUBSTANCE_SOURCE
	10-57299b68d4	MI-R1.3-57299b68d4-IN_VIVO_SUBSTANCE_SOURCE
**method/route of administration**	09-7a4d616c66	MI-R1.3-7a4d616c66-IN_VV_METHODS_ROA
	10-57299b68d4	MI-R1.3-57299b68d4-IN_VIVO_ADMINISTRATION_METHOD
	10-57299b68d4	MI-R1.3-57299b68d4-IN_VITRO_ADMINISTRATION_METHOD

	12-d2c4887a02	MI-R1.3-d2c4887a02-PROTOCOL_METHOD_OF_ADMINISTRATION
**exposure time**	03-88b52fa7b8	MI-R1.3-88b52fa7b8-EXPERIMENT_EXPOSURE_TIME
	09-7a4d616c66	MI-R1.3-7a4d616c66-IN_VV_METHODS_EXPOSE_TIME
	10-57299b68d4	MI-R1.3-57299b68d4-IN_VITRO_EXPOSURE_DURATION
	10-57299b68d4	MI-R1.3-57299b68d4-IN_VIVO_EXPOSURE_DURATION
**number of controls**	09-7a4d616c66	MI-R1.3-7a4d616c66-IN_VV_METHODS_CONTROLS
	10-57299b68d4	MI-R1.3-57299b68d4-IN_VIVO_NEGATIVE_CONTROLS
	10-57299b68d4	MI-R1.3-57299b68d4-IN_VITRO_POSITIVE_CONTROLS
	10-57299b68d4	MI-R1.3-57299b68d4-IN_VITRO_NEGATIVE_CONTROLS
	10-57299b68d4	MI-R1.3-57299b68d4-IN_VIVO_POSITIVE_CONTROLS
**stability**	08-3ce932c4f9	MI-R1.3-3ce932c4f9-STABILITY
	09-7a4d616c66	MI-R1.3-7a4d616c66-PCHEM_STABILITY
**Chemical composition**	01-75ec3968cc	MI-R1.3-75ec3968cc-INTRINSIC_CHEMICAL_COMPOSITION
	02-649848907b	MI-R1.3-649848907b-NM_COMPOSITION
	04-faf3eea67a	MI-R1.3-faf3eea67a-PCHEM_CHEMICAL_COMPOSITION
	08-3ce932c4f9	MI-R1.3-3ce932c4f9-COMPOSITION
	09-7a4d616c66	MI-R1.3-7a4d616c66-PCHEM_COMPOSITION
	11-1fb3ef13d0	MI-R1.3-1fb3ef13d0-PCHEM-COMPOSITION
	12-d2c4887a02	MI-R1.3-d2c4887a02-MAT-COMPOSITION_AND_SYNTHESIS
**Surface Chemistry/Coating/Functionalization**	01-75ec3968cc	MI-R1.3-75ec3968cc-INTRINSIC_SURFACE_COATINGS
	02-649848907b	MI-R1.3-649848907b-NM_SURFACE_FUNCTIONALIZATION

	06-324358ad68	MI-R1.3-324358ad68-I-SURFACE_CHEMISTRY
	08-3ce932c4f9	MI-R1.3-3ce932c4f9-SURFACE_CHEMISTRY
	09-7a4d616c66	MI-R1.3-7a4d616c66-PCHEM_SURFACE_CHEMISTRY
	11-1fb3ef13d0	MI-R1.3-1fb3ef13d0-PCHEM-SURFACE_CHEMISTRY
**Purity**	01-75ec3968cc	MI-R1.3-75ec3968cc-INTRINSIC_PURITY
	04-faf3eea67a	MI-R1.3-faf3eea67a-PCHEM_PURITY
	08-3ce932c4f9	MI-R1.3-3ce932c4f9-PURITY
	09-7a4d616c66	MI-R1.3-7a4d616c66-PCHEM_PURITY
	10-57299b68d4	MI-R1.3-57299b68d4-IN_VITRO_SUBSTANCE_PURITY
	10-57299b68d4	MI-R1.3-57299b68d4-IN_VIVO_SUBSTANCE_PURITY
**Crystallinity**	01-75ec3968cc	MI-R1.3-75ec3968cc-INTRINSIC_CRYSTALLINITY
	09-7a4d616c66	MI-R1.3-7a4d616c66-PCHEM_CRYSTALLINITY
**Surface Area**	01-75ec3968cc	MI-R1.3-75ec3968cc-INTRINSIC_SURFACE_AREA
	04-faf3eea67a	MI-R1.3-faf3eea67a-PCHEM_SURFACE_AREA
	08-3ce932c4f9	MI-R1.3-3ce932c4f9-SURFACE_AREA
	09-7a4d616c66	MI-R1.3-7a4d616c66-PCHEM_SURFACE_AREA
	11-1fb3ef13d0	MI-R1.3-1fb3ef13d0-PCHEM-SURFACE_AREA
**Surface charge**	01-75ec3968cc	MI-R1.3-75ec3968cc-EXTRINSIC_CHARGE
	03-88b52fa7b8	MI-R1.3-88b52fa7b8-PARTICLE_SURFACE_CHARGE
	04-faf3eea67a	MI-R1.3-faf3eea67a-PCHEM_SURFACE_CHARGE
	08-3ce932c4f9	MI-R1.3-3ce932c4f9-SURFACE_CHARGE

	09-7a4d616c66	MI-R1.3-7a4d616c66-PCHEM_SURFACE_CHARGE
	11-1fb3ef13d0	MI-R1.3-1fb3ef13d0-PCHEM-SURFACE_CHARGE
**Solubility**	04-faf3eea67a	MI-R1.3-faf3eea67a-PCHEM_SOLUBILITY
	07-1582738113	MI-R1.3-1582738113-PCHEM-SOLUBILITY
	08-3ce932c4f9	MI-R1.3-3ce932c4f9-SOLUBILITY
	11-1fb3ef13d0	MI-R1.3-1fb3ef13d0-PCHEM-SOLUBILITY
**Shape**	01-75ec3968cc	MI-R1.3-75ec3968cc-INTRINSIC_SHAPE
	02-649848907b	MI-R1.3-649848907b-NM_SHAPE
	03-88b52fa7b8	MI-R1.3-88b52fa7b8-PARTICLE_SHAPE
	08-3ce932c4f9	MI-R1.3-3ce932c4f9-SHAPE
	09-7a4d616c66	MI-R1.3-7a4d616c66-PCHEM_SHAPE
	11-1fb3ef13d0	MI-R1.3-1fb3ef13d0-PCHEM-SHAPE
	12-d2c4887a02	MI-R1.3-d2c4887a02-MAT-SIZE_SHAPE_DIMENSIONS
**Size**	01-75ec3968cc	MI-R1.3-75ec3968cc-INTRINSIC_SIZE_DISTRIBUTION
	02-649848907b	MI-R1.3-649848907b-NM_SIZE_DISTRIBUTION
	03-88b52fa7b8	MI-R1.3-88b52fa7b8-PARTICLE_SIZE_DIAMETER
	03-88b52fa7b8	MI-R1.3-88b52fa7b8-PARTICLE_ASPECT_RATIO
	04-faf3eea67a	MI-R1.3-faf3eea67a-PCHEM_SIZE_DISTRIBUTION
	04-faf3eea67a	MI-R1.3-faf3eea67a-PCHEM_ASPECT_RATIO
	08-3ce932c4f9	MI-R1.3-3ce932c4f9-SIZE
	08-3ce932c4f9	MI-R1.3-3ce932c4f9-SIZE_DISTRIBUTION

	09-7a4d616c66	MI-R1.3-7a4d616c66-PCHEM_SIZE_DISTRIBUTION
	09-7a4d616c66	MI-R1.3-7a4d616c66-PCHEM_SIZE
	11-1fb3ef13d0	MI-R1.3-1fb3ef13d0-PCHEM-PARTICLE_SIZE
	12-d2c4887a02	MI-R1.3-d2c4887a02-MAT-SIZE_SHAPE_DIMENSIONS
**Zeta potential**	01-75ec3968cc	MI-R1.3-75ec3968cc-EXTRINSIC_CHARGE
	12-d2c4887a02	MI-R1.3-d2c4887a02-MAT-ZETA_POTENTIAL
**Dispersibility**	03-88b52fa7b8	MI-R1.3-88b52fa7b8-PARTICLE_POLYDISPERSITY
	11-1fb3ef13d0	MI-R1.3-1fb3ef13d0-PCHEM-DISPERSIBILITY
**Organism/Species (in-vivo)**	06-324358ad68	MI-R1.3-324358ad68-I-COLLECTION_ORGANISMS
	09-7a4d616c66	MI-R1.3-7a4d616c66-IN_VIVO_SUBJECT_SPECIES
	10-57299b68d4	MI-R1.3-57299b68d4-IN_VIVO_SPECIES
**Strain (in-vitro)**	09-7a4d616c66	MI-R1.3-7a4d616c66-IN_VIVO_SUBJECT_STRAIN
	10-57299b68d4	MI-R1.3-57299b68d4-IN_VIVO_ORGANISM_STRAIN
**Number of replicates**	02-649848907b	MI-R1.3-649848907b-MEASUREMENT_REPLICATES
	06-324358ad68	MI-R1.3-324358ad68-I-REPLICATES
	10-57299b68d4	MI-R1.3-57299b68d4-IN_VITRO_NUMBER_OF_REPLICATES
**data analysis method**	10-57299b68d4	MI-R1.3-57299b68d4-IN_VITRO_STATISTICAL_METHODS
	10-57299b68d4	MI-R1.3-57299b68d4-IN_VIVO_STATISTICAL_METHODS
	12-d2c4887a02	MI-R1.3-d2c4887a02-PROTOCOL_DATA_ANALYSIS_DETAILS
**Dose/Concentration**	02-649848907b	MI-R1.3-649848907b-MEDIA_NM_CONCENTRATION
	03-88b52fa7b8	MI-R1.3-88b52fa7b8-EXPERIMENT_DELIVERED_DOSE

	03-88b52fa7b8	MI-R1.3-88b52fa7b8-EXPERIMENT_ADMINISTRATED_DOSE
	09-7a4d616c66	MI-R1.3-7a4d616c66-IN_VV_METHODS_DOSE
	10-57299b68d4	MI-R1.3-57299b68d4-IN_VIVO_DOSE
	10-57299b68d4	MI-R1.3-57299b68d4-IN_VITRO_DOSE
	12-d2c4887a02	MI-R1.3-d2c4887a02-PROTOCOL_ADMINISTERED_DOSE
	12-d2c4887a02	MI-R1.3-d2c4887a02-PROTOCOL_DELIVERED_DOSE
